# Dual-Component Gelatinous Peptide/Reactive Oligomer Formulations as Conduit Material and Luminal Filler for Peripheral Nerve Regeneration

**DOI:** 10.3390/ijms18051104

**Published:** 2017-05-21

**Authors:** Caroline Kohn-Polster, Divya Bhatnagar, Derek J. Woloszyn, Matthew Richtmyer, Annett Starke, Alexandra H. Springwald, Sandra Franz, Michaela Schulz-Siegmund, Hilton M. Kaplan, Joachim Kohn, Michael C. Hacker

**Affiliations:** 1 Institute of Pharmacy, Pharmaceutical Technology, Leipzig University, 04317 Leipzig, Germany; caroline.kohn@uni-leipzig.de (C.K.-P.); annett.starke@uni-leipzig.de (A.S.); alexandra.springwald@uni-leipzig.de (A.H.S.); schulz@uni-leipzig.de (M.S.-S.); 2 Collaborative Research Center (SFB-TR67), Matrixengineering Leipzig and Dresden, Germany; sandra.franz@medizin.uni-leipzig.de; 3 New Jersey Center for Biomaterials, Rutgers, The State University of New Jersey, Piscataway, NJ 08854-8066, USA; bhatnagard@dls.rutgers.edu (D.B.); Woloszyn@bu.edu (D.J.W.); mdr154@scarletmail.rutgers.edu (M.R.); kaplanh@dls.rutgers.edu (H.M.K.); 4 Boston University School of Medicine, Boston University, Boston, MA 02118, USA; 5 Department of Dermatology, Venereology and Allergology of Medical Faculty of Leipzig University, 04317 Leipzig, Germany

**Keywords:** cross-linked gelatin, nerve regeneration, filler, maleic anhydride, oligomer, bioconjugation, LM11A-31, shear thinning, injectable hydrogel, human adipose-tissue derived stem cells

## Abstract

Toward the next generation of nerve guidance conduits (NGCs), novel biomaterials and functionalization concepts are required to address clinical demands in peripheral nerve regeneration (PNR). As a biological polymer with bioactive motifs, gelatinous peptides are promising building blocks. In combination with an anhydride-containing oligomer, a dual-component hydrogel system (cGEL) was established. First, hollow cGEL tubes were fabricated by a continuous dosing and templating process. Conduits were characterized concerning their mechanical strength, in vitro and in vivo degradation and biocompatibility. Second, cGEL was reformulated as injectable shear thinning filler for established NGCs, here tyrosine-derived polycarbonate-based braided conduits. Thereby, the formulation contained the small molecule LM11A-31. The biofunctionalized cGEL filler was assessed regarding building block integration, mechanical properties, in vitro cytotoxicity, and growth permissive effects on human adipose tissue-derived stem cells. A positive in vitro evaluation motivated further application of the filler material in a sciatic nerve defect. Compared to the empty conduit and pristine cGEL, the functionalization performed superior, though the autologous nerve graft remains the gold standard. In conclusion, LM11A-31 functionalized cGEL filler with extracellular matrix (ECM)-like characteristics and specific biochemical cues holds great potential to support PNR.

## 1. Introduction

Nerve transplants (autograft or allograft) or bioengineered artificial nerve guidance conduits (NGCs) are necessary to bridge peripheral nerve injures larger than 5 mm [1,2]. In the last decades, several “off-the shelf” NGCs based on type I collagen (Neuragen^®^, Neuroflex^TM^, NeuraWrap^TM^), poly(glycolic acid) (Neurotube^®^) or poly(d,l-lactide-co-ε-caprolactone) (Neurolac^®^) have been developed to meet the clinical performance of autologous nerve grafts [3,4]. As NGCs still fail to provide a protective environment in larger defects (>3 cm) over the entire healing process, it has become necessary to define standardized criteria for optimal NGCs design [3,4]. Several theories were presented to describe peripheral nerve regeneration (PNR) and to determine factors that distinctly contribute to axonal elongation from the proximal stump and growth of non-neuronal supporting cells [5]. It became evident that axonal reinnervation is attributed to the secretion of growth-promoting soluble factors from the distal end and the direct interaction to insoluble matrix substrates (i.e., topographical cues) [6,7]. Furthermore, this elongation requires permissive migration, coalescence and myelination of Schwann cells (SC), presence of SC-derived microtubes and the formation of cylindrical basement membranes as a characteristic of peripheral nerves [5,8,9]. Considering the different determinatives and stages of PNR, it is believed that microtubular guidance and low circumferential compression onto the regenerating nerve determine the overall clinical outcome in addition to diffusion of neurotrophic factors and presentation of topographical cues [5,10]. Consequently, bioengineered NGCs can support PNR (e.g., axonal elongation, nerve trunk maturation, regulation of tissue capsule, and microtube formation) by their pristine chemical composition, degradation rate, permeability, and by integration of growth-permissive factors and neurogenic cells [5]. As a natural, biodegradable polymer, gelatin can be used for NGCs fabrication, whereby the negligible content of aromatic amino acids entails low antigenicity and cytotoxicity while conserved bioactive motifs induce and promote PNR [11,12,13]. As gelatin undergoes a sol-gel transition, cross-linking with, e.g., genipin or carbodiimide, has been established to increase mechanical stability and prolong biodegradation of gelatin-based NGCs [11,12]. Besides the design of single-lumen conduits, gelatin can also be used for the design of a luminal filler [14,15]. In general, the filling concept of hollow NGCs aims at providing artificial endoneurial structures as physiological pathways for axonal regeneration in order to support intraluminal cell migration and bands of Büngner formation [16,17,18]. As natural extracellular matrix (ECM) and diverse biopolymers contribute to these processes, different luminal fillers derived from collagen, laminin, fibrin, alginate, and chitosan as well as gelatin have been proposed in the formation of longitudinally aligned fibers, porous sponges and hydrogels [1,19,20]. Besides filler properties (e.g., surface texture, porosity, chemical composition, mechanics), it is known that the presence of growth supporting factors and cells is beneficial [1,21]. In that regard, luminal fillers potentially combine multiple functionalities not only as a ECM-like guidance cue and haptotactic element but also for the delivery of neurotrophic cells and immobilized biochemical factors such as nerve growth factor (NGF) [1,22,23]. As a homodimeric neurotrophin, NGF binds to receptors such as sortilin (type-1-receptor), tropomysine kinase receptors and neurotrophin 75 receptors (p75^NTR^) and regulates the development, maintenance and functionality of the nervous systems [24,25]. For p75^NTR^ it is known that the receptor is re-activated in the adult nervous system following cellular stress and nerve injury which in turn activates co-receptors, such as tyrosine kinase receptors, and independent signaling pathways towards cell migration, survival and death [26,27,28]. Though the role of p75^NTR^ in proapoptotic and prosurvival pathways is controversially discussed and dependent on other parameters including intracellular adaptors, co-expressed receptors and homodimerization, it is hypothesized that the re-expression of p75^NTR^ in SC plays a major role in axonal growth and remyelination after nerve injury [26,28]. Based on that, neurotrophin active regions within NGF were identified to design simple non-peptide molecules that circumvent deleterious sequences but mimic monovalent NGF-p75^NTR^ binding region and eliminate the apoptotic influence of immature proNGF [29,30,31]. Among several NGF-hairpin loop 1 mimetics termed LM11A, the small molecule LM11A-31, also known as C31, was identified as a promising p75^NTR^ ligand potentially regulating the migration and differentiation of SC and remyelination [24,28,30,31,32]. In this study, a dual-component hydrogel system (cGEL) based on the cross-linking reaction between free amine functionalities in the collagen-derived peptide mixture Collagel^®^ (COL) and maleic anhydride (MA) in the oligomeric building blocks (oPNMA; for full name and chemical composition, refer to Section 3.2) was used in two bioengineering approaches, conduit and filler, for improved PNR [33,34,35]. In the first part, a recently described continuous dosing and templating process was utilized for the fabrication of cGEL-derived conduits (cCEL_conduit_) in a range of composition to test for bending stiffness, in vitro degradation and assess basic biocompatibility following subcutaneous (s.c.) implantation [34]. As current cGEL_conduit_ compositions still fall short of tensile strength to resist compression or collapse as well as suturability, cGEL filler formulations (cGEL_filler_) were developed and characterized. A shear thinning cGEL_filler_ was designed based on the cross-linking reaction between COL and GEL with oPNMA to be pre-gelatinized and subsequently injected into hyaluronic acid (HA) coated tyrosine-derived polycarbonate braided conduits (BC) [34,36].

Besides cGEL_filler_ as a pristine guidance matrix, the biochemical functionalization of the filler was realized by covalent immobilization of the small molecule LM11A-31. Based on our previous studies, LM11A-31 was reacted with a fraction of oPNMA anhydrides prior to the gelation reaction with collagen-derived peptide fractions [33,34]. The derivatization efficiency of oPNMA with LM11A-31 was examined and pristine cGEL_filler_ as well as LM11A-31-modified cGEL_filler_^+LM11A-31^ materials were mechanically characterized. The cytocompatibility of both fillers and the growth permissive effect of LM11A-31 were assessed in vitro with human adipose tissue-derived stem cells (hASC) prior to in vivo application in a rat sciatic nerve critically-sized defect model.

## 2. Results and Discussion

**Scheme 1:**
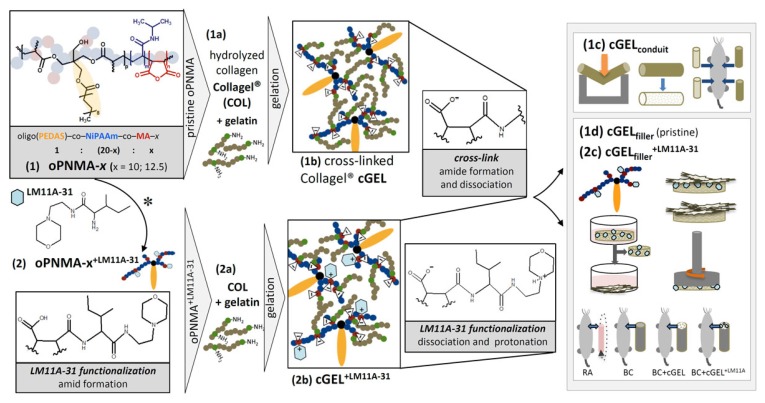
Graphical outline of hydrogel cross-linking, pre-derivatization and characterization of the hydrogel material as nerve guidance conduit and filler formulations. (**1**) Reactive building block oPNMA with two different maleic anhydride (MA) contents; (**1a**) cross-linking reaction of pristine oPNMA-x with free amine in Collagel^®^ (COL) (with or without additional gelatin) and the gelation base *N*-methylpiperidine-3-ol 20% (*v*/*v*); (**1b**) cross-linked hydrogel network (cGEL) with amide formation and dissociation after hydration to negatively charged networks; (**1c**) tube fabrication (cGEL_conduit_) with double syringe system and static mixing tip, material characterization of cGEL_conduit_ (from left to right: bending stiffness, in vitro degradation and *sub cutaneous* implantation in rat; and (**1d**) fabrication of pristine luminal filler (cGEL_filler_). (**2**) Pre-derivatization of oPNMA prior to gelation and incorporation of LM11A-31 to oPNMA-x with 2 equivalents of intact MA groups (MAeq = 2) (*) (oPNMA-x^+LM11A-31^), covalent binding by amide formation; (**2a**) cross-linking with COL and gelatin under base addition (‡) to cGEL_filler_^+LM11A-31^ with amide formation, dissociation and partial protonation of LM11A-31 residues to cationic moieties (**2b**); and (**2c**,**1d**) material characterization of cGEL_filler_ and cGEL_filler_^+LM11A-31^ (representative pictogram in right column, from left to right, top to bottom): derivatization capacity oPNMA-x, direct in vitro testing on cell growth of hASCs, indirect in vitro cytotoxicity testing of extracts on L929 fibroblasts, oscillation rheology, in vivo study in rat sciatic nerve model with reversed autograft (RA), empty braided conduit (BC), BC with pristine cGEL (BC^+cGEL^), and BC with cGEL^+LM11A-31^ (BC+cGEL^+LM11A^).

A graphical outline of the multifunctional anhydride-containing oligomeric building block oPNMA, pre-derivatization of oPNMA, hydrogel cross-linking to pristine and functionalized cGEL, and the characterization of cGEL materials as NGCs and cGEL_filler_ is provided in Scheme 1.

### 2.1. CGEL_conduit_ for Peripheral Nerve Repair

As previously published, our dual component hydrogel system based on partially hydrolyzed collagen COL and the building block oPNMA has been formulated to produce elastic, free-standing three-dimensional constructs [34]. Material composition and fabrication method was adjusted to allow for controllable gelation kinetics and injection molding of monolithic and tubular designs. In this follow-up study, cross-linked hydrogel tubes (cGEL_conduit_) were further characterized. In both in vitro degradation and three-point bending experiments, selected tube formulations were investigated for effect of oligomer type and influence of gelation base on conduit properties. Conduits with different COL contents were implanted s.c. to monitor biocompatibility and in vivo degradation.

#### 2.1.1. Mechanical Characterization and In Vitro Degradation

**Figure 1:**
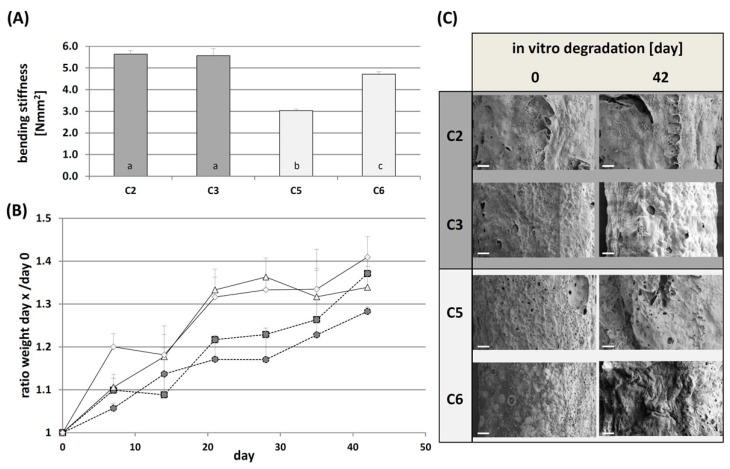
Mechanical properties and degradation of cross-linked hydrogel conduits cGEL_conduit_. (**A**) Bending stiffness of conduits after rehydration and equilibration at 37 °C for 30 min. Columns and error bars represent means ± standard deviation (*n* = 5). Means with different letters are statistically significantly different (*p* < 0.05); (**B**) In vitro degradation in PBS (pH = 7.4) at 37 °C for 42 days. Conduits fabricated from oligomeric building block oPNMA-10 (solid lines for C2 (circles) and C3 (square)) and oPNMA-12.5 (dashed lines for C5 (rectangle) and C6 (diamond)). Data points and error bars represent means ± standard deviation (*n* = 5); (**C**) Scanning electron microscopy (SEM) images of representative samples after 0 and 42 days of degradation. Scale bars represent 200 μm.

From previous work it was known that the reactivity of the two component COL/oPNMA formulations was dependent on the type of oligomeric cross-linker (i.e., MA content) and type and concentration of the gelation base [33,34]. A continuous small volume mixing setup in combination with low concentrations of gelation base *N*-methylpiperidin-3-ol (NMPO) allowed for the fabrication of robust and flexible cGEL_conduit_ from highly reactive oPNMA-10 or -12.5 and high concentrations of COL [34]. Three-point bending may provide meaningful data to estimate the in vivo performance in initial stages of NGCs material development [37]. Four conduit formulations C2, C3, C5, and C6 (Table 1) were analyzed (Figure 1A) and the small deviation of the obtained data illustrated good reproducibility of conduit fabrication. For oPNMA-10-derived conduits C2 and C3, the bending stiffness (EI) was higher than for oPNMA-12.5-derived tubes C5 and C6. For C2 and C3, no difference concerning the COL content and NMPO concentration during gelation was detected, whereby the higher NMPO concentration for C6 yielded stiffer conduits as compared to C5. In general, all tested cGEL_conduit_ showed higher bending modulus than data reported for single lumen conduits made of cross-linked collagen or chitosan [36,38]. In addition, no irreversible deformation was seen which underlined a promising material elasticity of the developed conduits. The in vitro degradation of cGEL_conduit_ C2, C3, C5, and C6 was monitored for six weeks (Figure 1B,C).

Conduit mass was determined weekly and conduit morphology was imaged by scanning electron microscopy (SEM) at Days 0 and 42. For all formulations, the weight increased during incubation due to swelling by less than 50% of the initial wet weight. This process was accelerated with time by ongoing bond cleavage leading to a looser hydrogel network and allowing more water to penetrate in to the gel bulk. This hydrolysis resulted in multiple changes of hydrogel polarity, contraction forces and network rigidity [39]. The wet weight of dry blotted samples increased steadily for all formulations, whereby oPNMA-10 derived tubes showed minor swelling extents than oPNMA-12.5. As the conduit stiffness decreased over time, the cellular response of dorsal root ganglia (DRG) neurons and SC in terms of cell adhesion, proliferation, spreading, and phenotypical behavior would presumably be affected. This assumption is based on studies showing that cell–matrix and cell–cell interaction of regenerating nerves is dependent on the substrate stiffness, whereby intermediate and softer surfaces seem to promote neurite outgrowth [40,41,42]. However, as the interplay between hydrogel stiffness, gel concentration and interfiber spacing determines the decisive force between neuronal growth cones and substrate matrix, it is difficult to predict the in vivo performance (i.e., neuronal elongation) and cellular response to the swollen material [43]. Importantly, none of the tubes collapsed during six weeks which would otherwise promote ingrowth of macrophages and fibroblasts in vivo and further accelerate NGCs degradation [44]. The corresponding SEM images at Days 0 and 42 revealed that the hydrogel matrix was distinctly bulged and profiled for C3 and C6, but no major mass defects were detected in all tubes (e.g., wall cracks, major cavities, leakage). This underlines the good hydrolytic stability of the tested dual component materials at this stage.

#### 2.1.2. *Sub Cutaneous* Implantation of cGEL_conduit_

**Figure 2:**
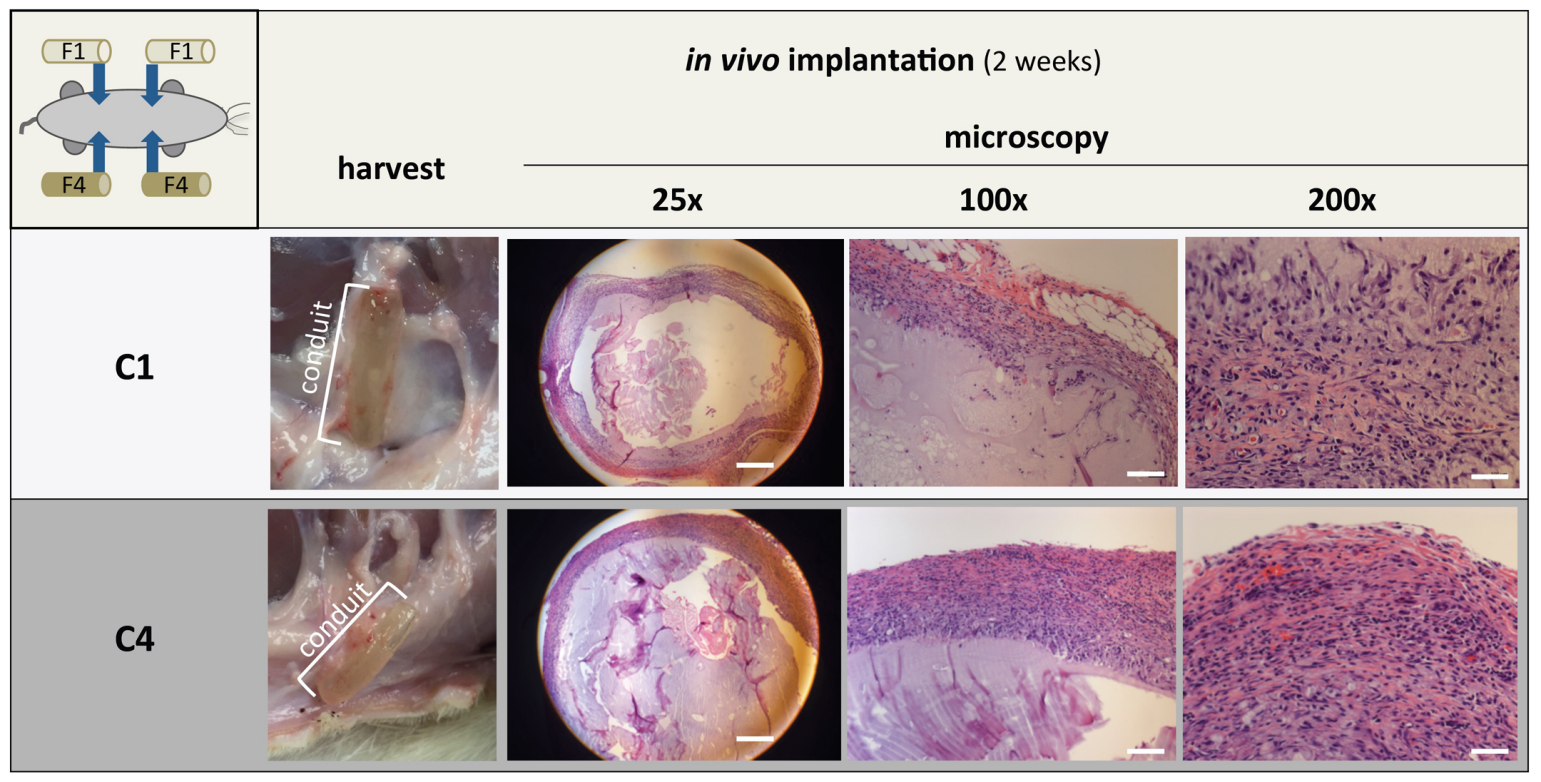
Subcutaneous implantation of conduits, formulation C1 and C4, for two weeks. Left column shows gross views of the implant upon harvest. The other columns show histological sections stained with hematoxylin and eosin (H&E) staining at different magnifications. Scale bars represent 200 μm.

In a previous study, we demonstrated that oPNMA and COL-based matrices were cytocompatible in direct contact in vitro experiments [34]. In the present study, s.c. implantation was performed to obtain in vivo data on cGEL_conduit_ degradation and biocompatibility. In detail, two oPNMA-10 derived formulations with low (C1) and high (C4) COL content and constant NMPO concentration were chosen. In these formulations, network density and rigidity was only influenced by the COL feed. For an intermediate COL concentration (15%), it was already seen that the cross-linking degree of composites fabricated by static mixing was above 80%, hence no pronounced material release due to insufficient cross-linking was expected to cause compatibility issues [34]. As biocompatibility is the result of a complex interplay of versatile cellular responses to chemical compounds, mechanical/biophysical properties and internalization mechanisms, the characteristics of the material as well as the host tissue have to be considered [45]. At this stage, C1 and C4 were implanted s.c. according to the standard of the International Organization of Standardization (ISO) 10993-6 and investigated for local effects. As expected, none of the animals showed severe rejection of the implanted conduits. After two weeks, conduits from both formulations were harvested. Some conduits had migrated inside the s.c. pocket. All explants were surrounded by a tissue capsule with minor vascularization (Figure 2). Histological analysis confirmed that the cells did not migrate through the conduit walls but formed condensed layers around the conduit which resulted in cGEL material degradation by cellular proteolytic activity from the outside to the inside of the conduit wall. Additionally, no considerable differences in the amount of granulomatous inflammation, including epithelioid macrophages, thick bands of fibrous connective tissue, mononuclear cell aggregates, neovascularization, and hemorrhage, were seen between C1 and C4. It is concluded that cGEL initiated a physiological, unspecific foreign body response, whereby the extent of inflammation varied predominantly within one conduit specimen. Thereby, the degree of inflammation was independent of the formulation, but differences between animals and implantation sides were observed. After six weeks, all implants were completely resorbed revealing that the in vivo degradation was faster than in vitro degradation due to enzymatic hydrolysis of the gelatinous peptide component (COL), probably stimulated by inflammatory cells. As no signs of pathological inflammatory reaction or scaring were seen, the low antigenicity and immunogenicity of gelatinous peptides due to the deficiency in immunogenic aromatic amino acids was proven [13]. Apparently, the oligomeric cross-linker component (oPNMA) did not impair material biocompatibility either. As artificial NGCs need to balance the criteria of mechanosensitive neuronal cells with anastomosis, in vivo wear and degradation, the conduit material requires further optimization regarding versatile mechanical compliance.

### 2.2. CGEL_filler_—Material Development and Characterization

The engineering of ECM-like luminal fillers strived to mimic the composition, morphology and regulatory function of natural intraluminal architecture. Such structures aim at supporting PNR in terms of guided cell migration, proliferation, differentiation, and secretion of trophic factors [5,21]. For the fabrication of a shear thinning hydrogel filler, a compromise between mechanical stiffness, shear thinning ability and biological activity was required. In our approach, the functionalities and physical properties of two types of gelatinous peptides (gelatin and COL) with the oligomeric building block oPNMA and the gelation base NMPO were formulated as cGEL_filler_: pristine (F1) and functionalized with LM11A-31 (F2) (Table 1). The filler materials were injected into established braided conduits from polymer fibers coated with HA [36].

#### 2.2.1. Filler Functionalization Concept

It is broadly described that growth factors such as NGF need to be immobilized and surmounted onto matrices or hydrogels to circumvent rapid and short-lived diffusion [22,23]. Based on the reported covalent binding capacity of MA groups in the oligomeric building block, the small nerve growth factor mimetic LM11A-31 was integrated into an injectable cGEL formulation (Scheme 1) [33,46]. The neurotrophic molecules LM11A have been designed as NGF-hairpin loop 1 mimetics with essential sequences reflecting the NGF-p75^NTR^-binding region [24,30,31,32]. As the p75^NTR^ ligand LM11A-31 potentially promotes migration and differentiation of SC towards effective remyelination, LM11A-31 was used as a promising biochemical cue in the filler [28,31,47]. Structurally, LM11A-31 offers positively charged groups and hydrogen bond donor as well as acceptor groups that may all contribute to NGF-like behavior by p75^NTR^ binding and/or disruption of relevant tertiary structures (Scheme 1) [30,31]. As a consequence of the covalent immobilization to oPNMA (oPNMA^+LM11A-31^), the primary amine in LM11A-31 was made unavailable for receptor binding. However, additional ionic groups were created by hydrolysis of residual MA, amide formation during cGEL formation and protonation of LM11A morpholine nitrogen. It is well established that positive surface charges introduced by polymeric coatings (e.g., poly-(l-)lysine) are beneficial for cell growth as they interfere with anionic cell membranes and promote cell attachment, spreading and growth [48,49]. We recently showed that positive moieties implemented in the negative cGEL network were cell-permissive and created focal adhesion sites for human sweat gland derived stem cells [34]. Therefore, it was assumed that LM11A-31 derived cationic moieties and anchoring sites enable contactattraction and chemoattraction of neuronal growth cones in the luminal filler [50].

#### 2.2.2. Quantification of LM11A-31 Derivatization

Direct quantification of filler derivatization with LM11A-31 was not possible. Different approaches were pursued to best estimate the derivatization degree and prove chemical immobilization to the cross-linker.

**Figure 3:**
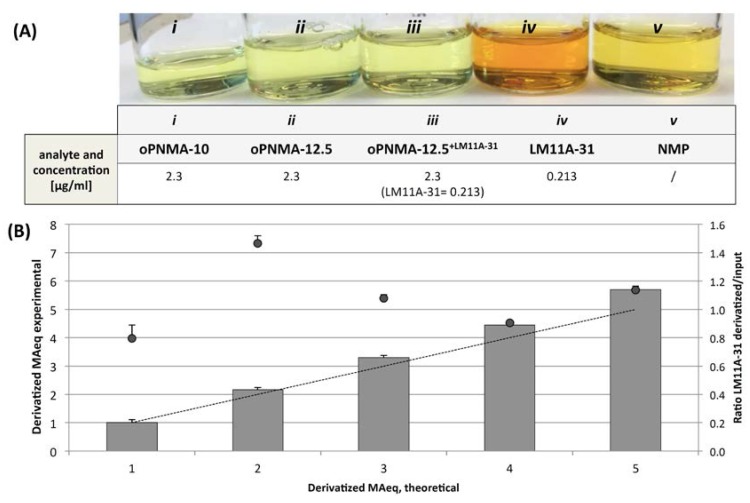
Colorimetric assay and indirect quantification of covalent derivatization of the oligomeric building block (oPNMA) with small molecule LM11A-31. (**A**) TNBS assay on pristine oPNMA solution, derivatized oPNMA^+LM11A-31^, free base LM11A-31, and solvent NMP. Analyte concentrations in depicted solutions were derived from precursor solutions as used for filler fabrication; (**B**) LM11A-31 quantification by secondary dansylcadaverine (Dcad) functionalization and subsequent chromatographic separation. LM11A-31 derivatization expressed as theoretical equivalents of intact MA functionalities (MAeq) relative to the quantified derivatized MAeq (experimental) in oPNMA-12.5 (bars). Dashed line indicates ideal correlation of theoretical (titration-based) and experimental values: Ratio of covalently grafted LM11A-31 to the amount of input (circles); columns, circles and error bars express sample means with standard deviation (*n* = 3).

**Scheme 2:**
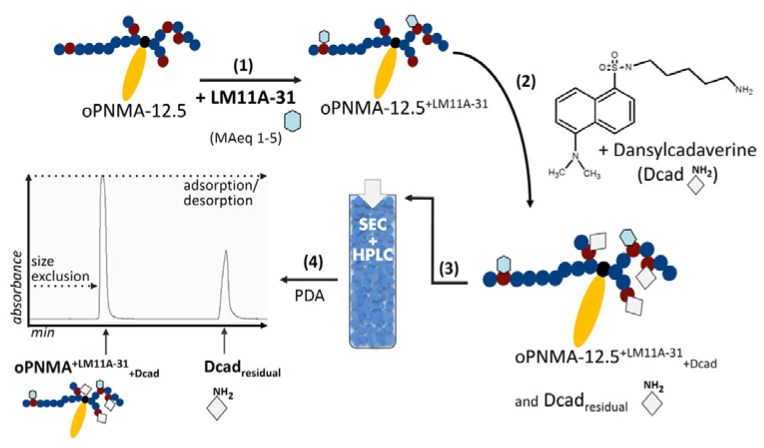
Schematic illustration of indirect quantification of covalent oPNMA derivatization with LM11A-31 by secondary Dcad functionalization and subsequent chromatographic separation. (**1**) OligoPNMA derivatization with 2 or 5 MAeq LM11A-31 to oPNMA-12.5^+LM11A-31^. (**2**) Derivatization of residual MA functionalities in oPNMA-12.5^+LM11A-31^ with excess amount of Dcad. (**3**) Chromatographic separation of residual Dcad and labeled oPNMA on porous column via size exclusion- and adsorption/desorption-driven separation. (**4**) Detection of residual Dcad by photodiode array detector and peak integration at 335 nm.

First, the covalent grafting of LM11A-31 onto oPNMA was investigated. A colorimetric staining based on the formation of a chromogenic, orange-colored derivative of primary amine with 2,4,6-trinitrobenzensulfonic acid (TNBS) was performed in solution. Pristine oligomeric solutions of oPNMA-10 and -12.5, LM11A-31, derivatized oPNMA^+LM11A-31^, and pure *N*-methylpyrrolidon (NMP) were tested in concentrations used in the filler formulations (Figure 3A). In both oPNMA solutions (*i*) and (*ii*), and NMP (*v*), no colorimetric changes were seen. The solution of LM11A-31 (0.213 μg/mL) was distinctly colored (*iv*). Afterwards, LM11A-31 was added to oPNMA-12.5/NMP (*ii*) in a concentration of 0.213 μg/mL equivalent to 2.5 MAeq (MAeq: quantity of derivatization expressed as equivalents of intact MA). As expected, oPNMA^+LM11A-31^ did not show a positive colorimetric shift due to complete conversion of all amine groups (*iii*). The detection limit of LM11A-31 with the TNBS assay was determined at 0.0017 μg/mL (data not shown). This let us assume that the covalent grafting of LM11A-31 to oPNMA was successful and almost quantitative. In a second approach, residual reactive anhydrides in oPNMA^+LM11A-31^ were functionalized with a predefined excess amount of dansylcadaverine (Dcad) yielding oPNMA^+LM11A-31^_+Dcad_ (Scheme 2).

Free Dcad was quantified from the derivatization solution by high performance liquid chromatography (HPLC) using porous size exclusion chromatography (SEC) column but combining the separation concepts of hydrodynamic size exclusion and adsorption/desorption. From preliminary work it was already suspected that the functionalization capacity of oPNMA, correlating with the content of chemically intact anhydrides per oligomer, was underestimated by the standard titration methods [33]. This observation was validated with this method, as it showed a binding capacity of Dcad to pristine oPNMA-12.5 solutions of more than 100% (based on titration data for chemically intact anhydrides). Based on chromatographic data, the actual content of chemically intact anhydrides was approximately 85% as compared to 71% determined by titrations (data not shown). Considering method specific error ranges, the true values are expected in between these results. This hypothesis was further assessed for the derivatization capacity of oPNMA-12.5 to LM11A-31 (Figure 3B). With increased input of LM11A-31 (MAeq = 1 to 5), the amount of incorporation exceeded theoretical values of anhydride groups derived from titration data. Additionally, the incorporation efficiency of LM11A-31 was indirectly calculated from Dcad co-grafting as the ratio of derivatized to input LM11A-31. As some values suggested efficiencies above 1 equivalent to 100%, interactions between the integrated LM11A-31 and the oligomeric chain were suspected. In detail, this was attributed to the protonated, positively charged tertiary nitrogen in the LM11A-31 morpholine ring upon anhydride cleavage and amide formation. As these moieties functioned as hydrogen donors, residual MA groups were cleaved and Dcad co-grafting reduced. In conclusion, the binding capacity of pristine oPNMA to small molecular amines was higher when monitored by this chromatographic method than what was expected from titration data irrespective of the organic solvent. Derivatization with LM11A-31 was very effective but sometimes exceeded theoretical values possibly due to ionic interactions and hydrogen shifts. However, taking into account the negative TNBS staining for oPNMA^+LM11A-31^ and low limit of detection it can be assumed that LM11A-31 was almost quantitatively grafted to oPNMA in a controllable manner.

#### 2.2.3. In Vitro Testing of LM11A-31 in Direct Contact with hASC

**Figure 4:**
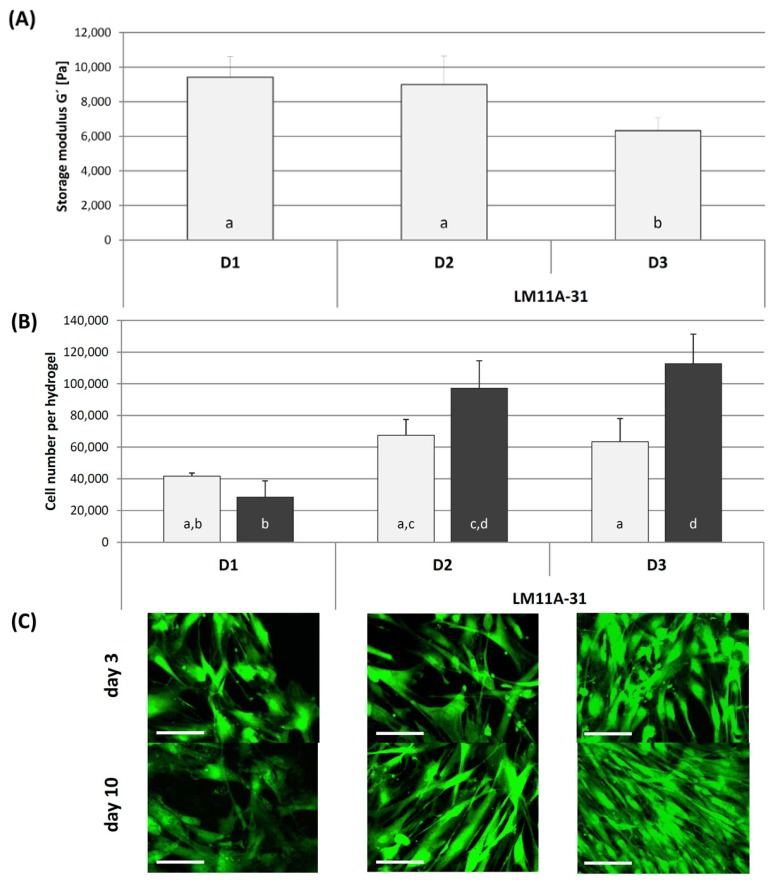
Direct cell contact of human adipose tissue-derived stem cells (hASC) on pristine and LM11A-31-derivatized cGEL_disc_. (**A**) Storage moduli G’ of hydrated cGEL discs at Day 0; (**B**) Cell number per hydrogel at Day 3 (light grey) and Day 10 (dark grey) on pristine cGEL discs (D1) and discs derivatized with 2.5 MAeq (D2) or 5 MAeq of LM11A-31. Storage moduli G’ of hydrated cGEL discs at Day 0 (empty circles). Columns with error bars represent means ± standard deviation (columns: *n* = 4; circles: *n* = 5). Means with different letters are statistically significantly different (*p* < 0.05); (**C**) Laser scanning microscopy micrographs (z stack of at least 20 micrographs) on Day 3 (first row) and Day 10 (second row). Scale bars represent 100 μm.

It has been described that morpholine moieties, as present in LM11A-31, can modulate cellular response, namely proliferation, metabolism, migration, and survival, by oxygen-hydrogen bonds [51]. In addition, as described earlier LM11A-31 specifically interacts with the NGF receptor in a selective manner. Therefore, promising effects of LM11A-31 modification of the cGEL materials (cGEL^+LM11A-31^) on adherent cells were expected not only for SC but for undifferentiated stem cells. To this end, hASC were cultivated for 3 and 10 days on sterilized pristine (D1) and LM11A-31 derivatized cGEL discs (cGEL_disc_), whereby two derivatization degrees were tested (MAeq 2.5 = D2 and MAeq 5 = D3). After Day 3, the number of cells on D2 and D3 was higher than on the pristine control (Figure 4B). This tendency was further pronounced after Day 10 and the number of cells was significantly higher on D2 and D3 than on D1, though no significant difference was monitored between D2 and D3 for both cultivation times. From laser scanning microcopy (LSM) images, it was evident that cells arranged in randomly scattered cell clusters on D1, whereas on both cGEL^+LM11A-31^ matrices, cell networks with elongated and aligned cell bodies were seen (Figure 4C). After Day 10, the cell layer was almost confluent on D3. Taken into account that no correlation between initial hydrogel stiffness, determined as storage modulus (G’) (Figure 4A), and cell number per hydrogel was seen, the enhanced cell adhesion and proliferation was attributed to the LM11A-31 functionalization. This assumption was supported by actin filament staining of hASC on D1 and D2 after Day 3 and Day 7 (see Supplementary Figure S1). On D2, the cytoskeleton was well oriented and elongated after Day 7, whereas this tendency was not seen on D1. On pristine D1, the cells remained randomly scattered and compact. Therefore, LM11A-31 allowed for directed cellular response of hASC as a surrogate marker for onward filler testing. In addition, hASC were reported to differentiate into neurospheres and SC-like cells with myelination ability which opens another niche for cGEL-based filler for cell delivery [12,52].

### 2.3. CGEL_filler_–Characterization, In Vitro and In Vivo Testing

#### 2.3.1. Shear Thinning and Micromechanical Characterization

**Figure 5:**
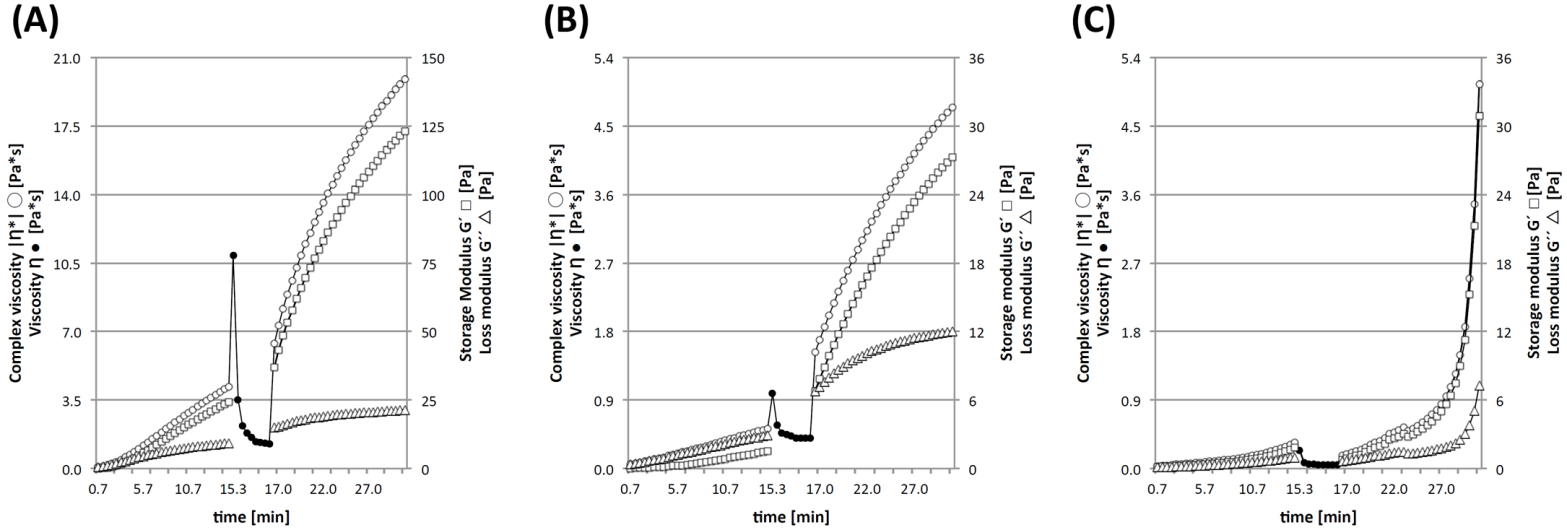
Rheological characterization of filling materials and control formulation. Gelation profile (0.7–14.7 min) displayed by storage modulus (G’, empty squares), loss modulus (G”, empty triangles) and complex viscosity (|η*|, empty circles); followed by shear thinning (15.5 to 16 min) displayed by viscosity (η, filled circles) and proceeding gelation (16.5 to 30.5 min) displayed by G’, G” and |η*|: (**A**) pristine cGEL_filler_ (F1); (**B**) derivatized cGEL_filler_^+LM11A-31^ (F2); and (**C**) uncross-linked Collagel^®^ and gelatin in the filler ratio as in F1 and F2. Data points represent average values (*n* = 5).

As in situ gelation is associated with various drawbacks, an ex vivo gelation prior to injection into the conduit lumen was aspired for NGCs filler material design [14]. For the design of pristine and derivatized cGEL_filler_ (F1 and F2), the gelation, shear thinning and post-shearing characteristics were analyzed. For F1 (Figure 5A), the storage modulus G’ increased almost linearly and gelation, defined as the cross-over of G’ and loss modulus (G”), was observed short of 4.5 min. Accordingly, the formulation complex viscosity (|η*|), increased with time. In the subsequent segment simulating shear thinning upon injection, system viscosity—here expressed as η because rotation and not oscillation was applied in this segment-rapidly decreased. During the following segement, the complex viscosity was restored within 0.5 min and G’ and |η*| increased further to values of 120 Pa and 20 Pa*s, respectively. It was thereby confirmed that the cross-linking reaction processed after shear thinning (e.g., upon injection) without disturbing hydrogel formation. For the derivatized filler F2 (Figure 5B), the parameters |η*|, G’ and G” were of lower values as compared to F1 and also increased steadily within the first section but gelation as cross-over of G’ and G” was not observed in this segment. The formulation F2 also showed shear thinning and ongoing gelation capacity after 17 min. In comparison, G’ observed for F2 was only 25% of G’ determined for F1 in this experimental set-up. The shape of the profiles of G’ and the gelation points indicate that chemical cross-linking and in the case of F2 integration of LM11A-31 via the derivatized oligomer can be assumed for these filler formulations. This is supported by the profile obtained for the COL/gelatin mixtures as found in the filler formulations (Figure 5C). In the initial period, less moduli increase was observed as compared to the formulations with cross-linker without cross-over of G’ with G”. This is attributed to the increase in viscosity of the gelatin fraction upon cooling on the rheometer. Following shear thinning, this process continued and physical gelation was observed. The profile differed from both filler formulations and the final values for G’ and η only reached 30 Pa and 5 Pa*s, respectively. Two shear thinning hydrogel fillers were formed with sufficient recovery/gelation capability that enabled safe and reliable injection and retention inside braided NGCs lumen under ambient conditions. With regard to the appropriate filler stiffness for nerve regeneration, it is well described that besides cross-linking degree and hydrophilicity, the hydrogel surface stiffness affects neuronal cell response [53,54]. In detail, DRG neurons sense soft and hard tissues and exhibit a narrow degree of mechanosensitivity [40]. Furthermore, the differentiation, migration, activity, proliferation, and survival of neuronal stem cells can be modulated by luminal rigidity [55]. Thereby, it has been reported that DRG neurons prefer materials with Young’s moduli around 1000 Pa or a storage modulus G’ between 60 and 200 Pa to support cell-specific force extension and cell movement [40,41,54,56]. SC, as the dominating glial cell type in the peripheral nerve system, also best responded to materials in the same stiffness range [40,42]. As a coplementary parameter, the effective Young’s modului (E*) of both filler formulations were determined with 93.6 ± 7.1 Pa (F1) and 64.1 ± 14.8 Pa (F2) by nanoindentation. Combining both material characteristics, it was assumed that the presented cGEL_filler_ formulations F1 and F2 have promising mechanical properties for further filler applications.

#### 2.3.2. Cytotoxicity Testing and Immunogenicity of cGEL_filler_ Composites

**Figure 6:**
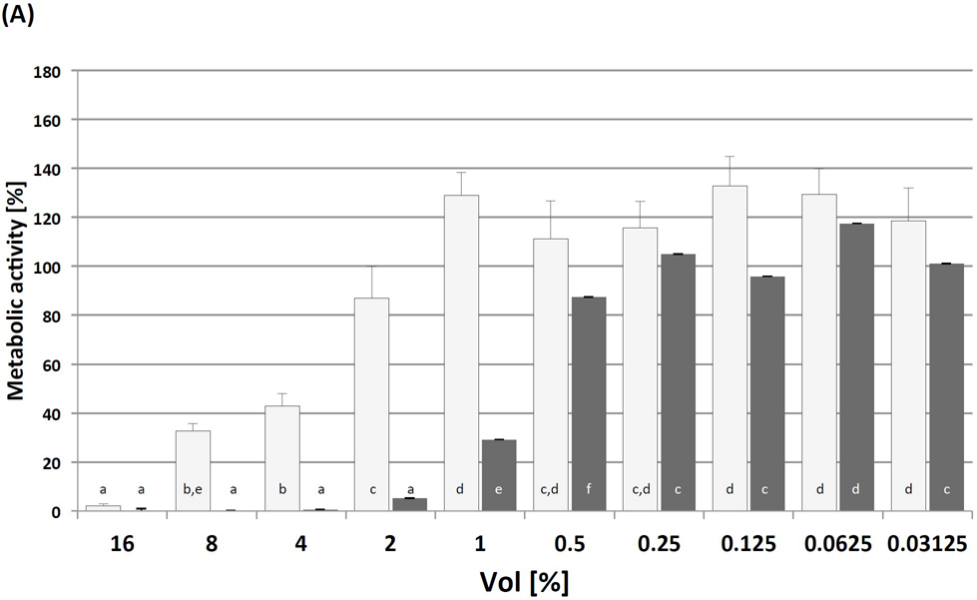

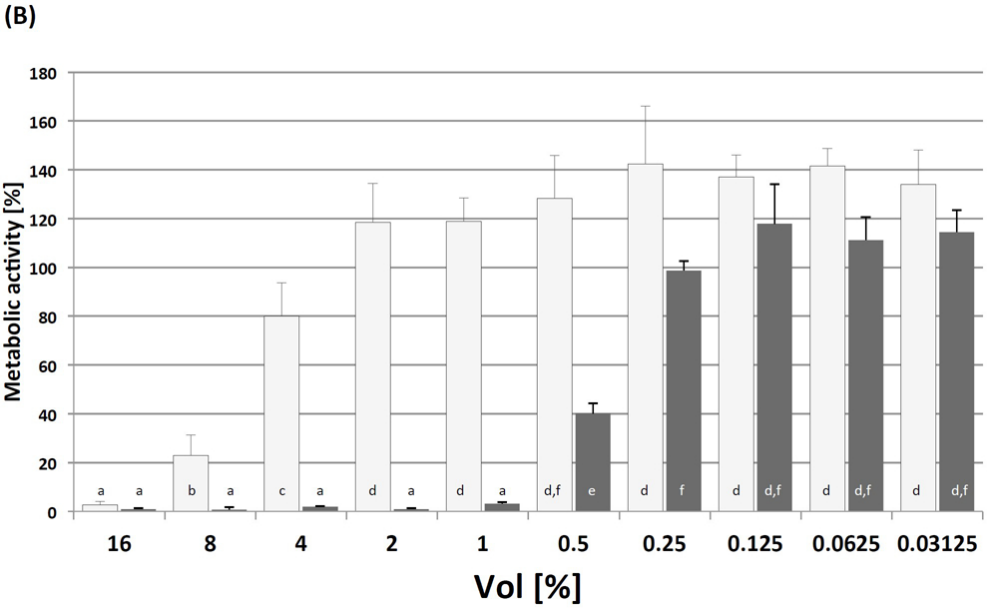

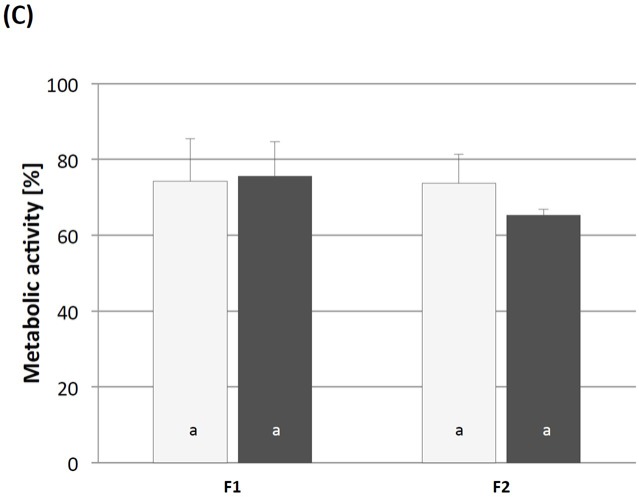
Cytotoxicity testing of: (**A**) NMP; and (**B**) NMPO in different volumetric fractions for 5 min (light grey) and 24 h (dark grey) incubation time in direct contact to hASC. Alamar Blue^®^ assay was performed 24 h after the incubation period was completed (**C**) Cytotoxicity testing of filler formulations using conditioned media and standard L929 fibroblasts. Cells were incubated with medium conditioned by filler formulations F1 and F2 for 24 h. Alamar Blue^®^ assay was performed immediately thereafter (24 h, light grey) and after 48 h of recovery in fresh cell culture medium (72 h, dark grey). For **A**–**C**: Cellular viability expressed as metabolic activity in relation to positive control groups (100% activity). Columns with error bars represent means ± standard deviation (*n* = 4). Columns with different letters are statistically significantly different (*p* < 0.05).

**Figure 7:**
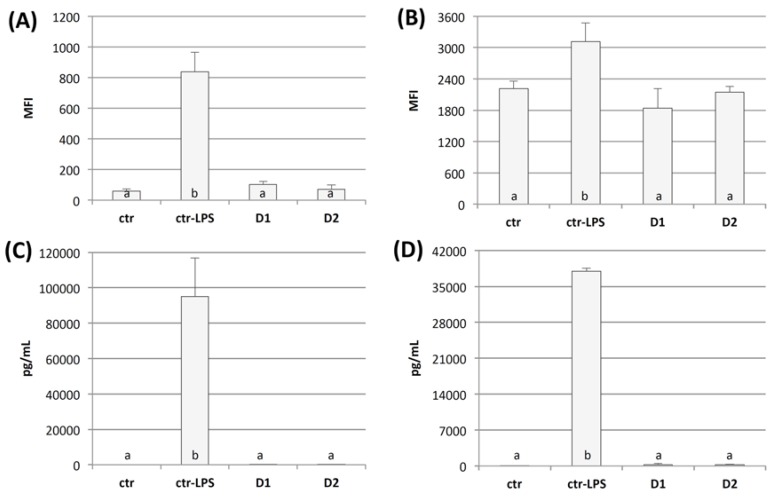
Immunogenicity of D1 and D2. The surface marker CD80 (**A**); and HLA-DR (**B**) by flow cytometry expressed as median fluorescence intensity (MFI); as well as the cytokines tumor necrosis factor (TNF) (**C**) and interleukin-12 (IL-12) (**D**) in the supernatant expressed as pg/mL. Columns with error bars represent means ± standard deviation (*n* = 3). Columns with different letters are statistically significantly different (for **A**, **C**, and **D**: *p* < 0.01; for **B**: *p* < 0.05).

Recently, we showed that cGEL_disc_ derived from oPNMA-10, COL and NMPO are non-cytotoxic on L929 mouse fibroblasts in diluted primary extracts [34]. In comparison to cGEL_disc_, the presence of NMPO and NMP in the injectable formulation was suspected to effect cytocompatibility, hence both components were tested individually on L929 fibroblasts in direct contact for different incubation times. NMP is listed in the United States Pharmacopeia/National Formulary (USP/NF) and the European Pharmacopeia and frequently used excipient for solubilization in parenteral applications as so called injectable solvent and for permeability enhancement in ophthalmic-drug-delivery-systems [57]. Though NMP is reported to show low acute toxicity, no genotoxicity and no carcinogenic effects, teratogenicity at higher levels has been reported [58]. Therefore, NMP is considered a class 2 residual solvent in the USP/NF. This implies that the amount of NMP should be limited in formulations or biomedical devices. The daily exposure should not exceed 530 ppm (0.053%) or 5.3 mg. Although the amount of NMP in F1 and F2 was twice as high as the daily exposure limit, the amount of NMP was necessary to combine the formulation components in solution. In contrast, the employed base NMPO is not listed in any of the aforementioned pharmacopeia and little information on NMPO cytocompatibility is available thus far. Both excipients were tested in direct contact with hASC for 5 min and 24 h by addition of different volume fractions. Afterwards, the medium was replaced with fresh cell culture medium and the metabolic activity was assessed after additional 24 h (Figure 6A–C) in relation to the positive control groups. For longer contact time (24 h), both substances showed considerable toxicity at all concentrations above 1% (NMP) and 0.5% (NMPO) indicated by relative metabolic activities below 70%. Especially for the piperidine derivative NMPO, this toxicity was notable despite the absence of chemical elements decisive for toxicity in structurally related alkaloids such as a carbon chain at the α-position to the nitrogen or a double bond between the α-carbon and the nitrogen [59,60]. For short contact time (5 min), the cellular metabolism was above 80% for concentrations below 4% (NMP) and 8% (NMPO). Regarding the long-term effect (after recovery of two days), cells maintained around 90% of their initial metabolism for NMP and NMPO below 1% irrespective of the initial contact time (data not shown). For all concentrations above, the metabolic reduction was independent of the initial contact time and also showed high recovery rates with metabolic activity above 100%. Based upon these results it was concluded that NMP and NMPO are non-cytotoxic on hASC at medium concentrations as low as 0.5% (NMP) and 0.25% (NMPO), irrespective of the initial contact time with relative cell viability above 70% according to ISO standard 10993-5. Regarding the cGEL_filler_ formulations F1 and F2, it was evident that the concentration during fabrication considerably exceeded the determined critical conditions. Consequently, in situ gelation of the fillers was not considered to eliminate the risk of precursor leakage and circumvent the effect of physiological conditions onto the gelation procedure. However, there remains a considerable concern on the compatibility due to NMP, NMPO and hydrogel precursor molecules. With regard to the high cross-linking degree determined for F1 (80.17 ± 2.39) and F2 (84.76 ± 2.37) above 80%, the amount of leachable hydrogel building blocks was low. In a following step, conditioned extract media were prepared from the F1 and F2 by placing different pre-gelled filler formulations in cell culture medium, diluted (1:10) and tested on L929 fibroblasts (Figure 6C) [13]. As the in vitro testing system was only static and not dynamic, the dilution step simulated the presence of body fluid in the surrounding tissue and physiological PNR clearing mechanism [61]. For F1 and F2, it was seen that after 24 h incubation the metabolic activity was above 70% though the extracts were not fully inert to L929 fibroblasts. Within 72 h after the initial exposure, cell metabolism was not significantly affected. Despite the monitored in vitro cellular response to F1 and F2, it was assumed that the in vivo diffusion rate within the braided conduit were sufficient to allow NMP and NMPO elimination as small molecular waste products [1,61,62]. Additionally, the physiological clearance of neuronal debris from macrophages, SC and revascularization would contribute to a physiological clearance after material insertion within a few days [16,61,63]. In that context the immunogenicity of D1 and D2 was tested on human macrophages whose inflammatory activation is vital for an effective immune response. Blood-derived monocytes were differentiated into inflammatory macrophages that present a pro-inflammatory activation profile in response to an activating stimulus, like lipopolysaccharide (LPS), similar to macrophages found at inflammation sites [64,65]. We thus assessed whether our cGEL formulation provoke a pro-inflammatory activation of the macrophages similar to stimulation with LPS, which may be critical for the onset of an immune response. For this, we seeded the inflammatory macrophages on the different cGEL_disc_ and used stimulation with LPS as a positive control for inflammatory activation on tissue culture plates. After cultivation for 24 h, macrophage activation was determined by analyzing the release of the inflammatory cytokines tumor necrosis factor (TNF) and interleukin-12 (IL-12) and the expression of the macrophage activation markers cluster of differentiation 80 (CD80) and human leukocyte antigen-D related (HLA-DR). The culture on both cGEL_disc_ D1 and D2 did not result in an up-regulation of macrophage activation markers or induction of cytokine release (Figure 7). A similar behaviour was seen for unstimulated macrophages in the negative control group. Contrary, LPS-stimulation of macrophages showed a high release of IL-12 and TNF as well as increased expression of CD80 and HLA-DR. These data demonstrate that the presented cGEL formulation D1 and LM11A-31 functionalization in D2 did not induce a pro-inflammatory activation of human inflammatory macrophages in vitro and suggest a low immunogenicity of the materials in vivo. As it is well described, such compendium of in vitro cytotoxic and immunogenicity testing relies on surrogate markers to quantify viable cells in an isolated test system. In consequence, the direct application in vivo was chosen to evaluate the effect of our novel biomaterial onto the regenerative nerve including the physiological environment and regenerative processes for rodents.

#### 2.3.3. In Vivo Testing of Luminal Fillers

**Figure 8:**
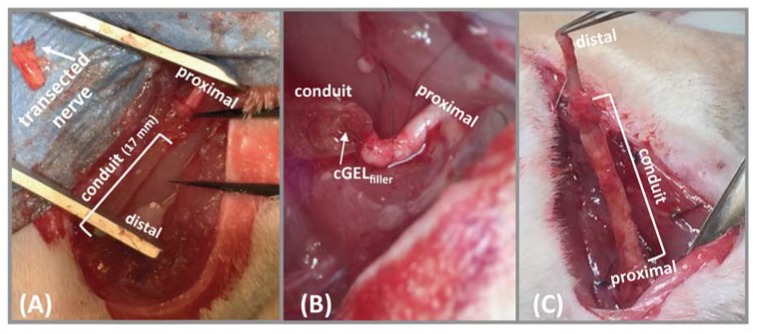
In vivo filler study in a critically sized rat sciatic nerve defect model. (**A**) Transection of 15 mm sciatic nerve, wound preparation and insertion of BC (17 mm length) with cGEL; (**B**) Suturing of the conduit through hydrogel filler and fixation on proximal and distal end; (**C**) Gross view of sample curing recovery after six weeks.

Braided conduits from tyrosine-derived polycarbonate with sufficient flexibility, mechanical strength and kink-resistance have been fabricated for PNR [36]. A hyaluronic acid coating was applied to yield a nanoporous conduit structure with positive influences on PNR [66,67]. These hollow tubes were used to house the filler formulations designed to function as ECM-like guidance cue and haptotactic element. Table 2 summarizes the implanted and tested groups. In Figure 8, the transection and insertion procedure is exemplarily depicted for group 3 (BC with cGEL_filler_, BC+GEL). In general, the areas of anastomosis and graft implantation were embedded in grafts were harvested from all animals and histologically evaluated.

**Figure 9:**
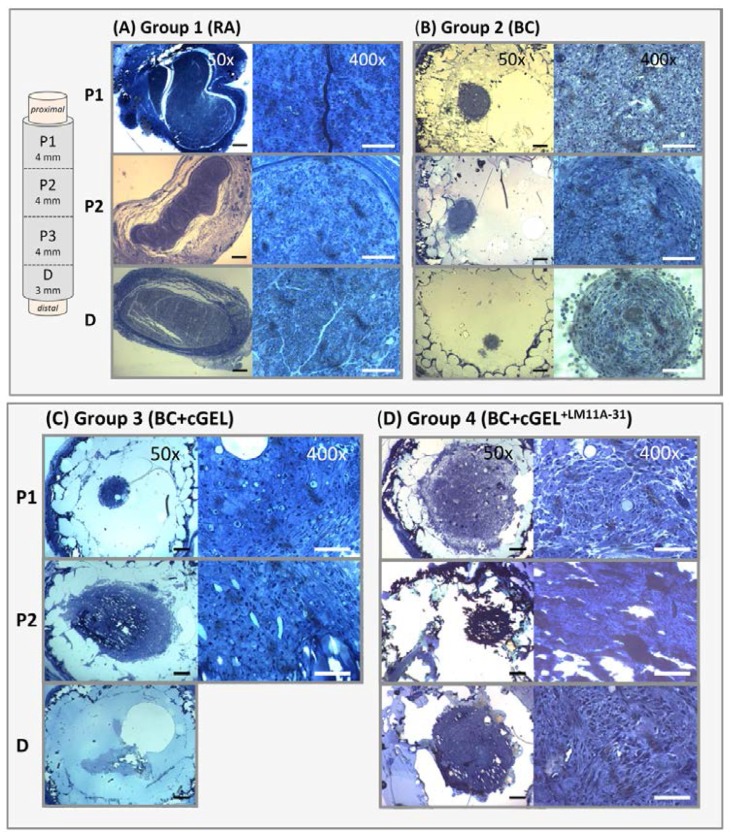
Histological images of transversal nerve segments after six weeks. The explanted graft was sectioned into four parts from proximal to distal end (P1, P2, P3, D) and P1, P2 and D were selected for histological processing from one representative animal per group. Transversal sections were stained with toluidine blue: (**A**) Reverse autograft (RA); (**B**) empty braided conduit (BC); (**C**) braided conduit with pristine cGEL filler (BC+cGEL); and (**D**) braided conduit filled with cGEL^+LM11A-31^ (BC + cGEL^+LM11A-31^). Scale bars represent 200 μm (black bars) and 50 μm (white bars) for 50× and 400× magnification, respectively.

Table 3 summarizes the results of the histomorphometric analysis of the first proximal section P1 of the regenerated nerve (Figure 9). The reverse autograft (RA) treated defect (group 1, RA) regenerated over a long distance to the distal end and exceeded the regeneration capacity of the empty braided conduits (group 2, BC), pristine filler (group 3, BC+cGEL) and LM11A-31 functionalized filler (group 4, BC+cGEL^+LM11A-31^).

This outcome strongly dependent on the number of recovered animals and, thereby, the animals that were included in the histomorphometric evaluation. As seen in Table 3, the number of included animals varied strongly that affected the statistical power of possible effects. In comparison to the gold standard RA, similar values in nerve cable area and axonal density were only found for BC+cGEL^+LM11A-31^, but nerve bundle organization and epineurium maturation have not been met, as nerve cables did not show tightly packed axons and fascicles. For BC, only three animals recovered, but nerve cable area and axonal density was only slightly lower as compared to BC+cGEL^+LM11A-31^. Furthermore, the G-ratio was measured as a functional index for myelination of the regenerated axons, whereby an ideal G-ratio is assumed between 0.6 and 0.7 [68,69]. Groups 1 and 4 reached these values in all animals, whereas, in Group 2, only three out of five animals met this criterion. As only one out of five animals recovered in Group 3, a reliable evaluation of this group was not possible. It is suspected that the ingrowth of fibrous tissue, the abovementioned clearing processes for debris removal and a foreign body response after the material insertion impaired the overall regeneration in Groups 2 and 3 [16,61,63]. Consequently, multiple factors require consideration in prospective approaches to fully evaluate the regeneration potential of luminal filling materials [36]. In conclusion, the current in vivo application showed that the dual integration of ECM-based cGEL_filler_ as guidance matrix with focal adhesion sites and the small NGF-mimetic LM11A-31 promote potential for improved PNR and exceeded the outcome of established single lumen braided conduits [36]. A follow up study will have to investigate the long-term performance and the electrophysiological outcome of PNR using this material [40,70].

## 3. Materials and Methods

### 3.1. Materials

Pentaerythritol diacrylate monostearate (PEDAS), *N*-isopropylacrylamide (NiPAAm), 2,2-azobis(2-methylpropionitrile) (azobisisobutyronitrile, AIBN), *N*-methylpiperidin-3-ol (NMPO), *N*-methylpyrrolidon (NMP), gelatin type A (from porcine skin, bloom 300 (G300)), 2,4,6-trinitrobenzenesulfonic acid (TNBS), (2S,3S)-2-amino-3-methyl-*N*-[2-(4-morpholinyl)ethyl] pentanamide dihydrochloride (LM11A-31·2HCl), phosphoric acid, and dansylcadaverine (Dcad) were purchased from Sigma-Aldrich (Seelze, Germany). Maleic anhydride (MA) and sodium hydroxide were obtained from Acros Organics (Geel, Belgium). Fractions of partially hydrolyzed gelatinous proteins, Collagel^®^ (type B, 11.5 kDa, viscosity (10%, 25 °C): 3.58 mPas, Lot # 895046, COL) was kindly provided by Gelita AG (Eberbach, Germany). *N*,*N*-Dimethylformamide (DMF), methanol HPLC grade and phosphate buffered saline (PBS) were purchased from AppliChem (Darmstadt, Germany). Double syringe delivery systems and static mixers with 8 windings and conical outlet geometry were purchased from Adchem (Wendelstein, Germany). Adchem also provided templating cannulas with outer diameter of 1.1 mm. Static mixers with 8 windings and luer-lock system were acquired from Medmix System AG (Rotkreuz, Switzerland). Sterican^®^ needles for oligomer synthesis (1.2 × 40 mm, 18 G × 1.5”) and tubular molding (0.80 × 40 mm, 21 G × 1.5”) were purchased from B. Braun Melsungen AG (Melsungen, Germany). Silicon tubes with an inner diameter of 2.5 mm and a wall thickness of 1 mm for tubular molding were obtained from Deutsch and Neumann (Berlin, Germany). HA coated braided conduits (length: 17 mm, inner diameter: 1.5 mm) were prepared as described previously [36]. For injection of the hydrogel filler into BC, Terumo™ insulin syringes 27 G × 1/2” (Terumo, Sommerset, NJ, USA) were used. Dulbecco’s modified eagle medium (DMEM) with low glucose and without phenol red was purchased from Sigma Aldrich (Seelze, Germany). Fetal bovine serum (FBS), penicillin/streptomycin (P/S), and trypsin were purchased from PAA Laboratories (Pasching, Austria). Alamar Blue^®^ reagent and Calcein-acetoxymethyl (calcein AM) was produced by Invitrogen (Darmstadt, Germany). For immunogenicity testing, Roswell Park Memorial Institute medium (RPMI), FBS, P/S, and glutamine were obtained from Biochrom (Berlin, Germany). Granulocyte-macrophage colony-stimulating factor (GM-CSF) was purchased as Leukine^®^ (Berlex Richmond, VA, USA). Tumor necrosis factor (TNF) was purchased from eBioscience (Frankfurt, Germany) and interleukine-12 (IL-12p40) from Biolegend (Aachen, Germany). Anti-CD80-FITC and anti-HLA-DR-APC and corresponding isotype controls-FITC, -APC were obtained from BD Biosciences (Heidelberg, Germany).

### 3.2. Oligomeric Cross-Linker Synthesis and Characterization

Oligomeric cross-linkers oligo(PEDAS-co-NiPAAm-co-MA) (oPNMA) with different MA contents were synthesized as previously published [46]. Briefly, co-monomer mixtures of PEDAS, NiPAAm and MA in tetrahydrofuran were copolymerized by free radical polymerization with AIBN as initiator at 60 °C under a nitrogen atmosphere. In constant molar ratio, 1 mol PEDAS was co-polymerized with 20 mol of the co-monomers NiPAAm and MA. The resulting oligomeric macromers were abbreviated as oPNMA-x with x being the molar ratio of MA in the co-monomer feed relative to PEDAS. The resulting oligomers were precipitated in diethyl ether and vacuum-dried to obtain the final macromer. The chemical compositions and molecular weight distributions were verified as reported [46]. The fraction of chemically intact anhydride functionalities in oPNMA was calculated from combined conductometric and Brown–Fujimori titrations [46]. For cGEL_conduit_ and cGEL_filler_ fabrication, the oligomers were diluted in DMF and NMP, respectively.

### 3.3. Functionalization of cGEL with LM11A-31

#### 3.3.1. Procedure

The capability of oPNMA derivatization has been reported in a straightforward procedure [33,34]. Briefly, oPNMA-10 and -12.5 were derivatized with a defined submolar ratio (relative to chemically intact anhydride groups) of primary amine-functionalities as present in LM11A-31 in an amidation reaction while residual anhydrides remained available for the cross-linking of gelatinous components COL and GEL. In a typical set-up, normalized equivalents of chemically intact anhydride groups (MAeq) in oPNMA were coupled with LM11A-31 to oPNMA^+LM11A-31^. MAeq 2, for example, expresses that LM11A-31 is added as a molar equivalent of the experimentally determined intact anhydride groups per oPNMA that equals 2 moles per theoretical MA groups in oPNMA, e.g., 10 in oPNMA-10. Therefore, the dihydrochloride of LM11A-31 was diluted in NMP, transferred into the free base by addition of base and reacted with oPNMA. The coupling reaction was maintained for 15 min under gentle shaking and ambient conditions.

#### 3.3.2. Colorimetric Staining

As a proof of concept, a colorimetric staining based on the presence of primary amines was performed with TNBS as described [33,34]. In detail, the staining was done on pristine oligomeric solutions oPNMA-10 and -12.5, free base LM11A-31, derivatized oPNMA^+LM11A-31^ (MAeq 2.5) and pure NMP. All analytes were treated with four parts water, one part sodium bicarbonate (4%) and one part aqueous TNBS solution (0.5%). Afterwards, samples were hydrolyzed in excess volume of 6 N HCl and the reaction was visually analyzed.

#### 3.3.3. Quantification 

For quantification of the derivatization capacity, a combination of size exclusion chromatography (SEC) and high performance liquid chromatography (HPLC) was used to differentiate between pristine and derivatized oligomers. In detail, a secondary derivatization agent dansylcadaverine (Dcad) was bound to residual anhydrides of oPNMA^+LM11A-31^ and residual Dcad was quantified. The analysis was performed with a single porous size exclusion column (Proteema 300 Å, 5 μM, 8 × 300 mm, PSS Polymer Standard Service, Mainz, Germany) in a liquid chromatography system consisting of an autoinjector (SIL-10ADVP), a pump (LC-20AD), a degasser (DGU-20A5R), and a diode array detector (SPD-M10AVP) (all from Shimadzu, Duisburg, Germany). The mobile phase consisted of methanol with x% phosphoric acid (85%) and y% sodium hydroxide (3 M) (pH 4). The absorption wavelength of Dcad (λ_abs_) was found to be at 335 nm. For sample preparation, 1 mg pristine oPNMA-12.5 or derived oPNMA^+LM11A-31^ (MAeq = 1, 2, 3, 4, and 5) was diluted in NMP and reacted for 2 h under light exclusion with excess Dcad (MAeq = 14) to oPNAM_+Dcad_ or oPNMA^+LM11A-31^_+Dcad_. For Dcad calibration, appropriate dilutions of Dcad in NMP were prepared to yield 1 μg to 1000 μg in the final analyte solution. Afterwards, the mobile phase was added to all analytes to yield 4 mL and the solutions were filtered through 0.45 μM syringe filter (Minisart RC4, Sartorius AG, Göttingen, Germany). Finally, 50 μL of the eluate were ejected onto the preheated column (40 °C) and the elution was performed at a constant gradient with a flow rate of 1 mL/min over 40 min. Peaks were detected with the photodiode array detector and evaluated with the software LabSolutions (Version 5.42 SP3, Shimadzu). All measurements were performed in triplicates.

### 3.4. Hydrogel Fabrication

#### 3.4.1. Conduits

CGEL_conduit_ were fabricated as previously described in a continuous injection-molding process [34]. Briefly, a double syringe system with static mixing tip was used to inject a cGEL mixture into a tubular molding template. Therefore, a molding device was constructed from a silicon tube (diameter: 2.5 mm) in which the inner templating cannula (diameter: 1.1 mm) was placed and centrally fixed. Afterwards, cGEL_conduit_ were dried and washed to extract NMPO and DMF, freeze-dried for storage and rehydrated in PBS immediately before use. Table 1 depicts the cGEL_conduit_ compositions as used in different applications.

#### 3.4.2. Discs

CGEL_disc_ were fabricated as previously described [34]. Briefly, oPNMA-10 (pristine or derivatized) and aqueous COL solution were vortexed in the presence of NMPO (20% (*v*/*v*) aqueous dilution) in a ratio of 4:4:1. For derivatized species, oPNMA-10 was functionalized with LM11A-31 (MAeq = 2.5, 5) as described above prior to gelation. Following gelation, cGEL_disc_ were dried, washed and punched into discs (11 mm in diameter). For storage, cGEL_disc_ were lyophilized and sterilized by γ-radiation (13.5–16.5 kGy). The concentration of oPNMA/oPNMA^+LM11A-31^, COL and NMPO in D1 and D2 are depicted in Table 1.

#### 3.4.3. Filler Fabrication and Injection

Hydrogel filler formulations (F1 and F2) were fabricated with a double syringe delivery system and static mixing device [34]. Pristine oPNMA-10 was used for cross-linking of a premixture containing COL and GEL (both preheated to 37 °C) as well as NMPO to yield cGEL_filler_ (F1). Similarly, derivatized oPNMA-12.5 with 2.5 MAeq of LM11A-31 (oPNMA^+LM11A-31^) was used for fabrication of cGEL_filler_^+LM11A-31^ (F2). The concentration of oPNMA, COL, gelatin, and NMPO found in F1 and F2 are depicted in Table 1. For cytocompatibility testing and in vivo application, all pre-cursor solutions were filtered prior to mixing (0.22 μM syringe filter) and the hydrogel was manually injected through a static mixer into 1 mL syringe. The mixture was allowed to pre-gelatinize in the syringe at ambient conditions in all cases. For in vivo testing, 20–30 μL of cGEL_filler_ were injected the lumen of an empty BC that was pre-hydrated for 1 h using PBS. Excess cGEL_filler_ was removed from the ends during surgery prior to insertion and fixation to the nerve stumps. For ex vivo testing, cGEL_filler_ was injected into a testing vessel and allowed to relax for 30 min prior to testing.

### 3.5. Characterization of Cross-Linking and cGEL Hydrogel

#### 3.5.1. Mechanical Characterization of cGEL_disc_

For rheological characterization, cGEL matrices equilibrated in PBS were punched into smaller discs (diameter: 8 mm). The discs were measured by oscillation rheology equipped with an 8 mm steel plate at 37 °C under normal force control (0.2 N). During a frequency sweep (0.1 to 10 Hz) with a displacement of 0.17 mrad G’, G” and η* were recorded. G’ values were reported at a frequency of 1 Hz as an average from five samples.

#### 3.5.2. Gelation Profile and Shear Thinning of cGEL_filler_

The cross-linking reaction of oPNMA or oPNMA^+LM11A-31^ with COL and gelatin was analyzed by oscillation rheology. The rheometer (Physica MCR 301, Anton Paar, Graz, Austria) was equipped with a 25 mm steel cone (1°). For analysis of F1 and F2, a premixture of aqueous COL, gelatin (both at 37 °C) and NMPO was spread on the base plate (thermostatted at 20 °C) and the equivalent volume of oPNMA solution was pipetted on top. For the control group of uncross-linked COL and gelatin, the fraction of cross-linker and base was substituted by water to yield equivalent concentrations as in F1 and F2. In all cases, the gelation reaction was instantly induced by mixing of the components via a 720° rotation of the geometry within 30 s. Afterwards, the gelation profile was monitored for 15 min at a frequency of 1 Hz and a displacement of 0.17 mrad and storage modulus (G’), loss modulus (G”) as well as complex viscosity (|η*|) were recorded. Then, a dynamic shear was applied to simulate dosing of the filler from a syringe. During this segment the viscosity (η) was recorded while the shear rate was linearly increased from 10 to 250 s^−1^ over 30 s and maintained at 250 s^−1^ for another 15 s. Subsequently, the recovery/continued gelation of the formulation was monitored for 15 min under oscillation like in the first segment at 1 Hz and a displacement of 0.17 mrad. The experiments were performed in quintuplicate.

#### 3.5.3. Micromechanical Characterization of cGEL_filler_

The micromechanical properties of filler formulations F1 and F2 were obtained with a Piuma Nanointenter (Optics11, Amsterdam, The Netherlands). The measurement system, kindly provided by Optics11, introduces a new technology that makes use of a cantilever-based fiber-optical interferometry technology. The formulations F1 and F2 were freshly fabricated by static mixing and 800 μL filler was ejected into a culture dish (diameter: 35 mm). The material was hydrated in excess PBS and shortly equilibrated. The hydrogel surface was indented at different spots and single indentation measurements were performed using an indentation probe with a spherical tip (cantilever stiffness: 0.028 N/m). The load-indentation curves were recorded and the Hertzian contact model (at 60% of the loading curve) was used to calculate the effective Young’s Modulus according to the method provided [71]. Thereby, the Poisson ratio is excluded for calculation of the effective Young’s Modulus (E*) and E* is derived from the slope of the unloading section of the stress-strain curve, indenter tip radius and the final indentation. Detailed calculations can be taken from the literature [72,73]. The indentation depth was about 13 μm during each indentation test in different areas of the sample. The number of areas for F1 and F2 were 9 and 5, respectively. All measurements were performed at room temperature.

#### 3.5.4. Cross-Linking Degree

A standard TNBS assay was performed to determine residual free amines in cGEL_filler_ as reported [33,34]. Briefly, 5 mg of lyophilized cGEL_filler_ samples were immersed in the TNBS reaction mixture and incubated as described above. For blank values, HCl was added prior to TNBS. Reference values were obtained from a blend of COL and gelatin in the filler ratio of approximately 5:1. The absorbance of all solutions was measured at 340 nm (Cary^®^ 50 UV-Vis, Varian Inc, Palo Alto, CA, USA) and the cross-linking degree (CLD) was calculated according to Equation (1) [74].

(1) $$\mathrm{CLD}\;(\text{\%})=\left(1-\left(\frac{\mathrm{absorbance}\;\mathrm{cGELfiller}}{\mathrm{absorbance}\;\mathrm{COL}\text{\_}\mathrm{gelatin}}\right)\right)$$

Data was recorded in quadruplicate.

#### 3.5.5. Bending Stiffness of cGEL_conduit_

Three-point bending tests were performed with MTS Sintech 5/D Universal Testing Machine (MTS System Corporation, Minneapolis, MN, USA) equipped with 10 N load cell. Hydrated, 15 mm conduits were incubated at 37 °C for 30 min prior to testing and placed on the supporting pins of the three-point bending apparatus set to a distance of 1 cm. The loading pin was lowered at a crosshead speed of 10 mm/min. From the force versus elongation data, the bending stiffness (EI) of different cGEL_conduit_ formulations was determined by using the Equation (2):

(2) $$\mathrm{EI}=\frac{F}{d}\;\left({\frac{L}{48}}^{3}\right)$$

with F/d as the slope of the linear region in the force versus displacement plot and L is the distance of the support pins (10 mm) [36,38]. All measurements were performed in quintuplicate.

#### 3.5.6. In Vitro Degradation of cGEL_conduit_

Conduits of formulation C2, C3, C5, and C6 (*n* = 5) as depicted in Table 1 were UV irradiated for 45 min and hydrated with PBS under aseptic conditions. The conduits were trimmed to 10 mm length and weighted (initial wet weight). Afterwards, single cGEL_conduit_ were transferred into sterile Eppendorf Tube™ and covered with 5 mL sterile PBS followed by static incubation at 37 °C and 5% CO_2_. For weight measurements (wet weight) after Days 7, 14, 21, 28, 35, and 42, cGEL_conduit_ were botted dry on the surface, transferred into sterile weighing chambers, weighted, returned into the storage tubes and freshly incubated with PBS to a constant filling level. At Days 0 and 42, hydrated samples were examined microscopically, subsequently freeze-dried for 24 h (Benchtop Manifold Freeze-Drier, Millrock Technology, Kingston, NY, USA) and imaged by scanning electron microscopy (Field Emission SEM, Zeiss Sigma 300, 20 KV) after sputter coating with Au/Pd (SCD 004 sputter coater, 30 milliamps for 120 s).

### 3.6. In Vitro and In Vivo Compatibility

All in vivo experiments were performed at Comparative Medicine Resources (Rutgers, The State University of NJ, Piscataway, NJ, USA) in accordance with the published protocols of Rutgers Animal Care and Facilities Committee and the Institutional Animal Care and Use Committee (IACUC). All cell culture experiments were performed according to Helsinki guidelines, in compliance with national regulations for the experimental use of human material. 

#### 3.6.1. Subcutaneous Implantation of cGEL_conduit_

To access the biocompatibility of cGEL_conduit_, a six week s.c. implantation study was performed on Sprague—Dawley rats (weight 300–350 g). The anesthetized rats (isoflurane chamber) were shaved in their interscapular and lumbar region and four s.c. pockets per animal were created (1–1.5 cm). Hydrated cGEL_conduit_ of formulation C1 or C4 (Table 1) were inserted and closed using resorbable 5-0 sutures (Ethicon, Somerville, NJ, USA). Each rat obtained two conduits from each formulation. Animals were sacrificed after 2 and six weeks. Conduits were harvested, prepared for histological examination, cryosectioned, and stained with hematoxylin and eosin (H&E).

#### 3.6.2. Cytotoxicity of NMP and NMPO

The cytocompatibility test was performed according to ISO 10993-5 standard with hASC isolated with consent from informed healthy donors and used at passage 7. Cells were seeded (2500 per well) into 96 well plates (BD Falcon™) and maintained in culture medium (CM) composed of DMEM low glucose without phenol red enriched with 10% FBS and 1% P/S over night at 37 °C. Stock solutions of NMP and NMPO were prepared in CM, filtered through a 0.2 μM filter and diluted to yield concentrations of 16%, 8%, 4%, 2%, 1%, 0.5%, 0.25%, 0.125%, 0.0625%, and 0.03125%. The cells were incubated with these solutions (170 μL per well) for either 5 min or 24 h, washed with 200 μL PBS and incubated for 1 d with fresh culture medium before the Alamar Blue^®^ metabolic activity assay was performed [75]. Therefore, 10% reagent were added to each well and the reduction of resazurin (blue) to resorufin (red) by viable cells was quantified after 4 h at 37 °C under gentle shaking using a fluorescence plate reader (Infinite F 200, Tecan, Salzburg, Austria) equipped with a 570 nm/610 nm (excitation/emission) filter set. The fluorescence intensity was directly proportional to the number of viable cells and the metabolic activity in the extract groups was calculated relative to the live control. All measurements were performed in quadruplet.

#### 3.6.3. Indirect Cytocompatibility Test with cGEL_filler_ Extracts

The cytocompatibility test for filler F1 and F2 was performed according to ISO 10993-5 standard with L929 mouse fibroblasts (Cell Lines Service, Eppelheim, Germany) at passage 41 according to a published protocol [34]. The freshly prepared filler formulation was allowed to gel at ambient conditions before 180–200 μL of cGEL_filler_ were injected into wells of a 48 well plate and incubated over night at 37 °C. Then, 1 mL CM was added to each well and kept for 24 h at 37 °C in a humidified incubator under gentle shaking. Afterwards, the supernatant medium was aspirated, filtered (0.2 μM filter) and used as pristine primary extracts. Additionally, 1:10 dilutions with CM were fabricated as conditioned extracts. In parallel, L929 fibroblasts were seeded at a density of 10^4^ cells per 96-well and allowed to attach for 24h. The cell culture medium was replaced with 100 μL diluted extracts (*n* = 3) as conditioned medium or fresh cell culture medium for positive control. For a positive toxicity control, cells were treated with 70% (*v*/*v*) ethanol in water. After 24 h of incubation at 37 °C and gentle shaking, the metabolic activity was assessed using the Alamar Blue^®^ assay as described above and expressed relative to the values determined for the positive control groups (100%). Afterwards, all media were exchanged to fresh culture medium and the cells were kept for another 48 h at 37 °C under 5% CO_2_ atmosphere until the assay was repeated as described above (72 h values). All measurements were performed in quadruplet. 

#### 3.6.4. Immunogenicity of cGEL_disc_

Human peripheral blood was taken from healthy volunteers after approval of the local ethics committee (# ethic vote: 064-2009). CD14+ mononuclear cells were isolated from the blood by density-gradient centrifugation (Ficoll-Paque PLUS, GE Healthcare, Freiburg, Germany) and enrichment using the CD14+ Cell Isolation Kit (Miltenyi Biotech, Teterow, Germany) as previously described [76]. CD14 positive monocytes were seeded in RPMI supplemented with 1% (*v*/*v*) P/S, 1% (*v*/*v*) glutamine and 10% (*v*/*v*) FBS. Differentiation of monocytes towards inflammatory macrophages was induced by addition of 50 U/mL GM-CSF and cultivation for six days. Mature macrophages were harvested and seeded either on cGEL_disc_ or in two sets of controls on agarose-coated tissue culture plate. For the positive control set, macrophages were stimulated with LPS to trigger their inflammatory activation. In the second negative control set, macrophages remained unstimulated. After incubation for 24 h, macrophage activation was analyzed in all wells by assessing the release of inflammatory cytokine and the upregulation of activation markers. The levels of the inflammatory cytokines TNF and IL-12 were quantified by ELISA according to the manufacturer’s protocol in cell-free supernatants that were collected from the macrophage cultures. For surface marker expression, macrophages were harvested, washed and incubated with anti-CD80-FITC and anti-HLA-DR-APC and corresponding isotype controls -FITC, -APC (all from BD Biosciences, Germany) for 30 min at 4 °C. Antibody binding was measured by flow cytometry (Facs Canto II, BD Biosciences) and data analyzed using BD FACSDIVA™ software (BD Biosciences).

#### 3.6.5. Direct In Vitro Cell Contact to cGEL_disc_

Human ASC at passage 4 were seeded onto discs of formulation D1 and D2 (Table 1). Therefore, sterilized discs were rehydrated in 48 well plates with DMEM for 24 h at 37 °C. The medium was removed and sterilized silicone O-rings were inserted to maintain the disks submerged in medium. Afterwards, 500 μL cell suspension with 30,000 cells per gel (95 mm^2^) were added and incubated at 37 °C and 5% CO_2_. The medium was exchanged every other day and cell proliferation was assessed by Alamar Blue^®^ assay after Days 3 and 10 (*n* = 4) [75]. For visual examination, cGEL_disc_ (Days 3 and 10) were washed, incubated with PBS and stained with 2 μM calcein-AM prior to laser scanning microscopy (LSM 700, Zeiss, Oberkochen, Germany) with z-stack-imaging. Stacks were reconstructed from at least 20 individual micrographs.

### 3.7. CGEL_filler_ in a Rat Sciatic Nerve Injury Model

#### 3.7.1. Surgical Method and Animal Groups

In vivo nerve regeneration was evaluated in a critical size 15 mm rat sciatic nerve injury model for 6 weeks for 4 different groups (Table 2). Twenty Sprague-Dawley rats (weight 300–350 g) were anesthetized by placing them in an isoflurane chamber. The left sciatic nerve was exposed and the nerve was dissected 2 mm from the proximal end. Then a nerve segment (approximately 15 mm) was resected to create a 15 mm peripheral nerve defect. In reverse autograft groups (group 1), the 15 mm nerve segment were resected and sutured back in reverse orientation using 9–0 perineurial sutures (Ethicon, NJ, USA). For groups 2–4, the conduits were sutured to the distal and proximal stumps using the same suture material. The incised muscles and skin were closed using resorbable 5-0 sutures (Ethicon, NJ, USA).

#### 3.7.2. Histomorphometric Analysis

After 6 weeks, rats were euthanized using ketamine/xylazine anesthesia, the reconstructed sciatic nerve was exposed and fixed in-situ by immersion of the tissue in 4% formaldehyde. The conduits were divided into 4 segments (P1: 4 mm, P2: 4 mm, P3: 4 mm, D1 3 mm) from the proximal to the distal end. The samples were morphometrically analyzed after toluidine staining according to established protocols [36,68,77]. The overall number of axons per nerve cross-section (axonal density, axons/mm^2^), cross-sectional area of the regenerated cable (nerve cable area, μm^2^) and G-ratio (ratio of axon diameter to total fiber diameter) were analyzed in segment P1 using ImageJ. In addition the length of cable formation from the proximal end was assessed.

### 3.8. Statistics

Data were expressed as means ± standard deviations (SD). Single-factor analysis of variance in conjunction with Tukey’s Post Hoc test was performed to assess statistically significant differences (*p* < 0.05 and *p* < 0.01) within data sets.

## 4. Conclusions

In this study, the oligomeric cross-linker oPNMA and gelatinous-peptide COL were successfully used as dual-component materials (cGEL) in two different compositional approaches for continuous manufacturing of nerve guidance conduit and luminal filler. The presented cGEL-based NGCs were mechanically stable with comparable bending properties for GEL-based single-lumen NGCs. The combined data from in vitro and in vivo (s.c.) degradation revealed that the material swelling was limited and an open lumen was maintained. An accelerated in vivo degradation due to the physiological foreign body reactions was found. The implants were resorbed within six weeks and no specific immune reaction was visible which encourages further material development towards clinical application as single-lumen template. The dual-component cGEL was developed towards an injectable filler by changing building block contents and adding higher molecular weight gelatin. For improved bioactivity, the small NGF loop-mimetic LM11A-31 was covalently immobilized to the anhydride-containing oligomer and integrated into the filler. The efficient integration of LM11A-31 to oPNMA prior to gelation was shown by colorimetric staining and advanced liquid chromatography method combining size exclusion and absorption/desorption principles for separation. In direct cell–matrix contact with hASC, growth-permissive effects were seen upon LM11A-31 functionalization regardless of the derivatization extent. Two liquid additives of the filler formulations, the commonly used injectable solvent NMP and the hydrophilic base NMPO were tested for cytocompatibility in direct cell contact. Both substances showed critical toxicity above a threshold concentration and raised concern with the amount of these excipients in the filler formulations. However, the pre-gelled hydrogel network did not reveal major cytocompatibility or immunogenicity issues. As the mechanical properties of the pristine and derivatized filler were promising, both compositions were tested in a rat sciatic nerve model of critical defect size. In comparison to the empty conduit, the functionalized filler performed superior after six weeks and all animals recovered. Compared to the current gold standard, LM11A-31 proved to be a promising bioactive component for enhanced PNR. In conclusion, functionalized cGEL_filler_^+LM11A-31^ provided mechano-biochemical stimulation of PNR and will further be optimized for long-term treatment.

## Figures and Tables

**Table 1:** Summary of cross-linked gelatin based hydrogel (cGEL) and cGEL/gelatin formulations used in this study.

	oPNMA			COL (Gelatin)	NMPO	Mechanical Testing		In Vitro		In Vivo	
	*x*	(%) ^1^	+LM11A-31 (MAeq)	(%) ^1^	(%) ^2^	Three-Point Bending	Rheology	Degradation	Indirect/Direct Cell Contact	s.c.	Sciatic Nerve Model
**cGEL conduit**											
C1	10	3.5		12.5	4					+	
C2	10	3.5		17.5	4	+		+			
C3	10	3.5		20	2	+		+			
C4	10	3.5		20	4					+	
C5	12.5	3.5		17.5	2	+		+			
C6	12.5	3.5		17.5	4	+		+			
**cGEL disc**											
D1	10	3.5		15	2		+		+		
D2	10	3.5	2.5	15	2		+		+		
D3	10	3.5	5	15	2		+		+		
**cGEL filler**											
F1	10	2.5		5.3 (1)	2		+		+		+
F2	12.5	2.5	2.5	5.3 (1)	2		+		+		+

^1^ Weight percentage in final hydrogel conduit; ^2^ weight percentage during gelation; ^+^ analytical method was applied to analyze the corresponding formulation. Oligomeric building block (oPNMA); Collagel^®^ (COL); *N*-methylpiperidin-3-ol (NMPO).

**Table 2:** Summary of implanted groups.

Group	Conduit Material	Filler	Abbreviation	Number of Animals
**1**	reverse autograft	-	RA	5
**2**	braided conduit	-	BC	5
**3**		cGEL	BC+cGEL	5
**4**		cGEL^+LM11A-31^	BC+cGEL^+LM11A-31^	5

Reverse autograft (RA); braided conduit (BC); BC with pristine cross-linked filler (BC+cGEL); BC with functionalized cGEL (BC+cGEL^+LM11A-31^).

**Table 3:** Histomorphometric analyses of luminal filler in in vivo study.

Group			Histomorphometric Measurements				
Number	Material	Number of Animals Recovered	Number of Animals Considered (*n* out of 5)	Nerve Cable Area (mm^2^) ^‡^	Axonal Density (Axons/mm^2^) ^‡^	G-Ratio ^‡^	Nerve Cable Length from Proximal End (mm)
**1**	RA	5	5/5	2.36 ± 0.27 ^a^	9646 ± 1856 ^a^	0.64 ± 0.03 ^a^	15
**2**	BC	3	3/5	1.23 ± 0.06 ^b,c^	10,980 ± 2673 ^a^	0.63 ± 0.05 ^a,b^	4–8
			5/5	0.74 ± 0.60 ^b^	6588 ± 5764 ^a,b^	0.38 ± 0.31 ^a,b^	
**3**	BC+cGEL	1	1/5	0.10 ^‡^	483 *	0.78 ^‡^	12
			5/5	0.02 ± 0.04 ^d^	97 ± 193 ^b^	0.16 ± 0.3 ^b^	
**4**	BC+cGEL^+LM11A-31^	5	5/5	1.63 ± 0.18 ^c^	8153 ± 4731 ^a^	0.69 ± 0.04 ^a^	4–12

**^‡^** Values with different letters are statistically significantly different (*p* < 0.05). * Value for BC+cGEL 1/5 were not included in statistical evaluation.

## Supplementary Materials

Supplementary materials can be found at www.mdpi.com/1422-0067/18/5/1104/s1.

## Abbreviations

AIBN	2,2-azobis(2-methylpropionitrile)
λ_abs_	absorption wavelength of Dcad
EI	bending stiffness
BC	braided conduit
calcein-AM	calcein-acetoxymethyl
COL	Collagel^®^
cGEL	oligomeric cross-linked Collagel^®^
cGEL_conduit_	cGEL derived conduit
cGEL_disc_	cGEL derived disc
cGEL_filler_	cGEL derived filler
|η*|	complex viscosity
CD80	cluster of differentiation 80
CLD	cross-linking degree
CM	culture medium
LM11A-31	(2S,3S)-2-amino-3-methyl-*N*-[2,[2-(4-morpholinyl)ethyl] pentanamide
Dcad	dansylcadaverine
DMEM	Dulbecco’s modified eagle medium
DRG	dorsal root ganglia
E*	effective Young’s Modulus
ECM	extra cellular matrix
FBS	fetal bovine serum
GM-CSF	granulocyte-macrophage colony-stimulating factor
H&E	hematoxylin and eosin
HLA-DR	human leukocyte antigen-antigen D related
HPLC	high performance liquid chromatography
hASC	human adipose tissue-derived stem cells
HA	hyaluronic acid
IACUC	Institutional Animal Care and Use Committee
IL-12	interleukin-12
ISO	International Organization of Standardization
LPS	lipopolysaccharide
LSM	laser scanning microscopy
cGEL^+LM11A-31^	LM11A-31 functionalized cGEL
cGELfiller^+LM11A-31^	LM11A-31 functionalized filler cGEL
G’	storage modulus
G”	loss modulus
MA	maleic anhydride
MAeq	equivalents of intact maleic anhydride
p75NTR	neurotrophin 75 receptors
NGF	nerve growth factor
NGCs	nerve guidance conduits
NiPAAm	*N*-isopropylacrylamide
NMPO	*N*-methylpiperidin-3-ol
NMP	*N*-methylpyrrolidon
DMF	*N*,*N*-dimethylformamide
oPNMA	oligomeric cross-linker oligo(PEDAS-*co*-NiPAAm-co-MA)
oPNMA^+LM11A-31^	oPNMA covalently functionalized with LM11A-31
oPNMA^+LM11A-31+Dcad^	Dcad covalently bound to oligomer oPNMA^+LM11A-31^
P/S	penicillin/streptomycin
PEDAS	pentaerythritol diacrylate monostearate
PNR	peripheral nerve regeneration
PBS	phosphate buffered saline
RA	reverse autograft
SC	Schwann cells
SEM	scanning electron microscopy
SEC	size exclusion chromatography
SD	standard deviations
s.c.	subcutaneous
TNBS	2,4,6-trinitrobenzenesulfonic acid
TNF	tumor necrosis factor
USP/NF	United States Pharmacopeia/National Formulary
