# Supplementary material for: Dual-Component Gelatinous Peptide/Reactive Oligomer Formulations as Conduit Material and Luminal Filler for Peripheral Nerve Regeneration
Source: Int J Mol Sci. 2017 May 21;18(5):1104. doi: 10.3390/ijms18051104 (PMC5455012; doi:10.3390/ijms18051104)
Supplement: Supplementary file 1 [file ijms-18-01104-s001.pdf]

Supplementary Materials: Dual-component Gelatinous Peptide/ Reactive Oligomer Formulations as Conduit Material and Luminal Filler for Peripheral Nerve Regeneration

Caroline Kohn-Polster, Divya Bhatnagar, Derek Woloszyn, Matthew Richtmyer, Annett Starke, Alexandra H. Springwald, Sandra Franz, Michaela Schulz-Siegmund, Hilton Kaplan, Joachim Kohn, Michael C. Hacker

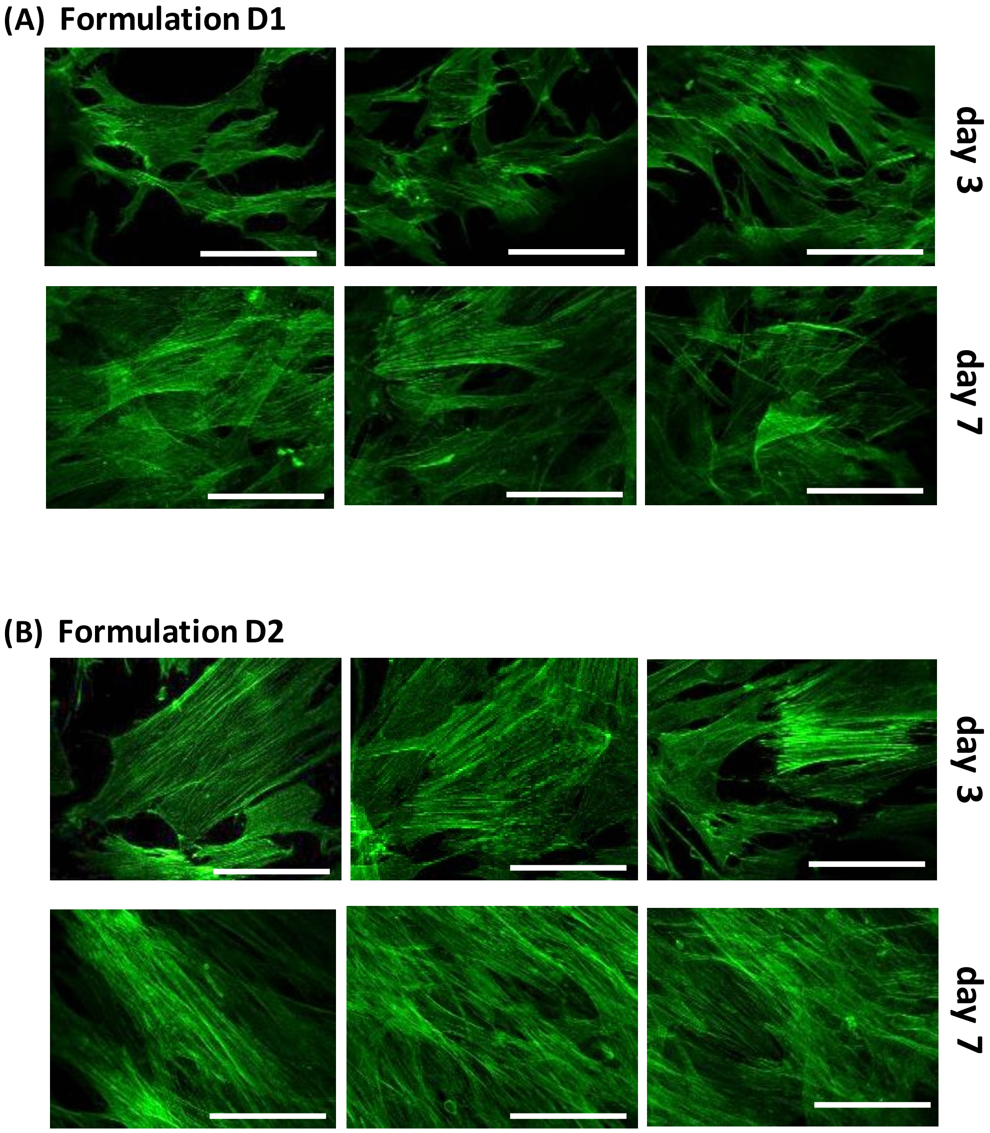

Figure S1. Direct cell contact of human adipose tissue-derived stem cells (hASC) on pristine and LM11A-31-derivatized discs of cross-linked hydrogel ($cGEL_{disc}$). Cells with a density of 10^4 cells/hydrogel disc were seeded on (**A**) oPNMA-10 (D1) and (**B**) oPNMA-$10^{+LM11A-31}$ (D2) derived hydrogel discs and stained after Day 3 (top row) and Day 7 (bottom row), the staining was performed with Alexa Fluor® 488 Phalloidin (Invitrogen™) according to the manufacture´s protocol; single stacks from lase scanning microscopy micrographs are depicted. Scale bars represent 100 µm.
